# Contribution of 16S rRNA gene PCR to microbiological diagnosis of endophthalmitis: a retrospective clinical cohort study of 71 vitreous and aqueous humor samples

**DOI:** 10.1186/s12348-026-00581-2

**Published:** 2026-04-25

**Authors:** Paul Robert, Sylvain Meyer, Fabien Garnier, Marie-Cécile Ploy, Pierre-Yves Robert, Maxime Rocher

**Affiliations:** 1 Ophthalmology Unit, Centre Hospitalier Universitaire de Limoges, 2 Avenue Martin Luther King, Limoges, 87042 France; 2 Bacteriology-Virology-Hygiene Laboratory, Centre Hospitalier Universitaire de Limoges, Limoges, France; 3 Inserm, RESINFIT, UMR 1092, Limoges, France

**Keywords:** Endophthalmitis, 16S rRNA gene, PCR, Bacterial identification, Ocular infection, Vitreous, Aqueous humor, Culture-negative

## Abstract

**Background:**

Culture remains the reference method for microbiological diagnosis of endophthalmitis, but its sensitivity is limited by small sample volumes, prior antibiotics, and fastidious organisms. This study evaluates the diagnostic contribution of 16S rRNA gene-targeted polymerase chain reaction (PCR) in improving bacterial identification in patients with endophthalmitis, compared to conventional culture.

**Methods:**

We conducted a retrospective cohort study including patients diagnosed with endophthalmitis and managed at Limoges University Hospital (France) from January 2018 to December 2024. Aqueous and/or vitreous samples were analyzed using both conventional culture and broad-range PCR targeting the bacterial 16S rRNA gene. The primary outcome was the global identification rate of pathogens by each method and their combination. Subgroup analyses considered the type of ocular sample.

**Results:**

Seventy-one intraocular samples from 50 episodes were analyzed (41 aqueous humor, 30 vitreous). Culture allowed bacterial identification in 21.1% of cases (15/71), compared to 39.4% for PCR (28/71, *p* = 0.002). The approach combining culture and PCR reached 42.3% (30/71, *p* < 0.001). Notably, PCR was positive in 26.8% of culture-negative cases (15/56), providing additional identification. Vitreous samples yielded higher identification rates than aqueous samples for both cultures (36,7% vs. 9,8%) and PCR (56,7% vs. 26,8%).

**Conclusions:**

PCR targeting the 16S rRNA gene significantly increases the diagnostic and the microbiological yield of intraocular sampling in endophthalmitis, particularly in culture-negative cases and in vitreous specimens. These findings support the complementary role of PCR in the microbiological workup of endophthalmitis.

**Supplementary Information:**

The online version contains supplementary material available at 10.1186/s12348-026-00581-2.

## Background

Endophthalmitis is a severe and potentially vision-threatening intraocular infection that can occur following exogenous inoculation such as intraocular surgery, intravitreal injections or open-globe trauma, or via hematogenous spread from distant infectious sites. Regardless of the etiology, whether exogenous or endogenous, the management of endophthalmitis relies on prompt initiation of appropriate antimicrobial therapy, ideally guided by microbiological identification of the causative pathogen [1]. Although culture remains the gold standard for microbial diagnosis of endophthalmitis, obtaining such identification can be challenging [2]. The sensitivity of conventional culture techniques is often limited by several factors, including the small volume of intraocular specimens, the tendency of microorganisms to adhere to intraocular lenses and capsular surfaces, resulting in low bacterial counts in aqueous and vitreous samples, prior antibiotic treatment, and the presence of fastidious or non-viable organisms [3]. Reported microbiological culture positivity rates vary between 20 and 40% for aqueous humor cultures and 30–50% for vitreous samples [4–7].

In recent years, molecular diagnostic tools have emerged to improve pathogen detection in intraocular infections. Among these, broad-range polymerase chain reaction (PCR) targeting the 16 S ribosomal RNA (rRNA) gene has shown promising results for the detection of bacterial DNA in minute ocular samples [8–11]. This method can detect the presence of DNA of dead organisms or free DNA and can provide a rapid identification in 48 to 72 h. Several studies have reported higher diagnostic yields with 16 S rRNA PCR, especially in patients previously exposed to antibiotics [4–7, 12–15]. PCR has also demonstrated utility in detecting bacteria that may not grow in conventional media, and in identifying pathogens in culture-negative cases where the clinical suspicion for infection remains high [12].

Despite these advantages, the implementation of 16S rRNA PCR in routine clinical practice remains variable, and its diagnostic performance in real-world settings is still under evaluation. In addition, most published studies have focused on specific subsets of endophthalmitis, particularly post-operative cases, and have not systematically assessed its performance across the full spectrum of etiologies, including endogenous forms.

In this retrospective cohort study, we aimed to evaluate the added diagnostic value of broad-range 16S rRNA gene PCR in comparison with conventional culture for microbial identification in patients with presumed infectious endophthalmitis, either exogenous or endogenous, treated at a tertiary care university hospital. By analyzing both aqueous and vitreous samples collected over a seven-year period, we sought to assess the global contribution of molecular diagnostics in clinical practice, particularly in culture-negative cases.

## Methods

### Study design and population

We conducted a retrospective, monocentric cohort study including all patients diagnosed with presumed infectious endophthalmitis at the University Hospital of Limoges (France) between January 2018 and December 2024, in whom both culture and 16S rRNA gene PCR were performed on at least one ocular sample (aqueous humor and/or vitreous). Both exogenous (postoperative, post-injection, post-traumatic, post-infectious keratitis) and endogenous endophthalmitis cases were included. Fungal cases were deliberately not excluded, as we aimed to evaluate the added value of PCR in real-life clinical conditions. Cases were identified through microbiology laboratory records and electronic medical charts.

The diagnosis of endophthalmitis was based on clinical signs indicative of intraocular infection involving both the anterior and posterior segments. Clinical features of anterior inflammation included periocular pain, decreased vision, conjunctival hyperemia, chemosis, corneal edema and inflammation in the anterior chamber (hypopyon, Tyndall, fibrin, cyclitic membrane). Inflammation of the posterior segment was confirmed either by the presence of vitritis on fundus examination or by ocular ultrasonography when media opacity precluded direct visualization.

Patients were excluded if no 16S rRNA gene PCR analysis was performed, if posterior segment inflammation was absent, or if the final diagnosis was deemed non-infectious after review of the clinical course and microbiological results.

### Sample collection and processing

Intraocular samples were collected at the time of diagnosis, before administration of the first intravitreal antibiotic injection. The choice of sampling site (aqueous humor, vitreous, or both) was left to the operating surgeon according to clinical presentation and technical feasibility. Aqueous humor was obtained by anterior chamber paracentesis, and vitreous samples were obtained via needle aspiration, outside the context of pars plana vitrectomy. All procedures were performed under aseptic conditions using standard ophthalmic surgical techniques. All eyes were sampled after topical anesthesia. Aqueous povidone–iodine 5% solution was instilled in the conjunctival sac after the lid speculum was placed, followed by irrigation with 20 mL of a sterile balanced salt solution before sampling. All samples were collected in a sterile syringe and immediately transferred to a DNA-free sterile container. The microbiology laboratory is located onsite, and samples were rapidly transferred via a pneumatic transport system. Because intraocular sample volumes were usually limited (< 300 µL), approximately 200 µL were allocated to molecular analysis by PCR and the remaining volume to conventional microbiological culture.

### Conventional microbiological culture

Samples intended for culture were promptly processed in the microbiology laboratory. Standard culture media were inoculated and incubated in different atmospheric conditions including a blood agar under anaerobiosis, a chocolate Polyvitex agar under CO2 enriched atmosphere and a thiol broth following French recommendations (REMIC v7.1). Plates were incubated for up to 5 days, with readings performed at 1, 2, and 5 days. Thiol broth was incubated for 5 days and systematically plated at day 5 to control the presence of bacterial growth in aerobic and anaerobic conditions. In the event of a positive culture, bacterial identification was carried out using standard MALDI-TOF mass spectrometry (Vitek^®^MS, BioMérieux, Marcy l’Etoile, France), and antimicrobial susceptibility testing was performed and interpreted according to the current recommendations at the time of the study, i.e. the European Committee for Antimicrobial Susceptibility Testing (http://www.eucast.org) guidelines [16].

### 16S rRNA gene PCR and sequencing

Total DNA was extracted from 200 µL of ocular samples using a commercial extraction kit (NucliSens easyMAG^®^, BioMérieux, Marcy l’Etoile, France). An internal control targeting the human β2-globin gene was used to control extraction process and absence of PCR inhibitors. A SYBR-Green qualitative PCR targeting a 492-bp fragment covering the V3-V4 region of the bacterial 16 S rRNA gene was performed using the 91E and 13BS primers previously described [17]. A PCR positive and a negative control as well as a negative extraction control were included. Amplification was performed on SmartCycler^®^ (Cepheid, Sunnyvale, CA, USA) and amplicons of positive samples were purified and sequenced on a 3500 XL (Applied Biosystem) Sanger sequencer. Sequence identity was determined by comparison with the GenBank database using the NCBI BLASTN tool. A sequence similarity ≥ 99% was considered indicative of species-level identification, and ≥ 97% for genus-level identification.

### Initial management and therapeutic strategy

All patients were hospitalized at diagnosis and received empirical systemic antibiotic therapy combining intravenous imipenem and levofloxacin. Intravitreal antibiotics consisting of vancomycin (1 mg/0.1 ml) and cefazolin (2.25 mg/0.1 ml) were administered immediately after sampling prior to microbiological results. These injections were repeated at 48 and 96 h according to clinical evolution. Adjustments to intravitreal therapy were made when microbiological results of culture and PCR testing were available. Intravitreal dexamethasone was added during the second and/or third injection series, only if clinical evaluation and/or culture and PCR results excluded a fungal etiology. Pars plana vitrectomy was considered in cases of unfavorable clinical evolution or lack of response to medical treatment. In cases of endogenous endophthalmitis with an identified systemic pathogen and available susceptibility testing, intravitreal antibiotic therapy was adapted to the causative organism in collaboration with the infectious diseases team.

### Data collection and outcome measures

Demographic, clinical, microbiological and therapeutic data were extracted from electronics medical records. The primary outcome was the diagnostic yield of conventional culture, 16S rRNA PCR, and their combination. Secondary outcomes included the additional diagnostic value of PCR in culture-negative cases, comparison of diagnostic yield between aqueous humor and vitreous samples, and concordance between methods.

### Visual prognosis assessment

To evaluate the association between pathogen identification and visual prognosis, a dedicated analysis was performed using residual best corrected visual acuity (BCVA) as the outcome. Residual BCVA was defined as the latest available measurement obtained after a minimum follow-up of six months, provided no intercurrent ocular event likely to affect vision had occurred. Cases with shorter follow-up were included only if final recorded visual acuity was no light perception with no expectation of recovery. All visual acuities were converted to the logMAR scale to allow statistical analysis (Additional file 1). For non-quantifiable values (e.g., hand motion, light perception), standard estimated logMAR equivalents were applied (Additional file 2).

### Statistical analysis

Descriptive statistics were used to summarize patient characteristics and microbiological results. McNemar’s test was used to compare the proportion of positive results between PCR and culture, and Fisher’s exact test was used to assess the association between visual acuity outcomes and microbial identification status. A p-value < 0.05 was considered statistically significant. Analyses were performed using Microsoft Excel (Microsoft Corporation, Redmond, WA, USA).

## Results

### Population characteristics

The study included 50 eyes from 50 different patients, with each case representing a single episode of presumed infectious endophthalmitis. No bilateral cases were observed. The cohort comprised 26 men (52%) and 24 women (48%) with a mean age of 71.1 years ± 15.6 (SD) years (range: 26–95 years). The right and left eyes were involved in 21 (42%) and 29 (58%) cases, respectively. Diabetes mellitus was present in 10 patients (20%). Initial BCVA was quantifiable in only 7 eyes (14%). A total of 9 (18%) eyes had BCVA limited to counting fingers, 25 (50%) perceived only hand motion, 8 (16%) had only perception of light, and one (2%) had no light perception at presentation (Additional file 3).

### Etiology and clinical context

The cohort included 18 cases of acute postoperative endophthalmitis (≤ 3 weeks after surgery) and 4 cases of delayed-onset postoperative endophthalmitis (> 3 weeks), as well as endogenous endophthalmitis (*n* = 10), post-intravitreal injection (*n* = 9), open-globe injuries (*n* = 4), post-infectious keratitis (*n* = 3), and cases of unknown origin (*n* = 2, referred to our tertiary center without available prior clinical records). Acute postoperative endophthalmitis occurred after cataract surgery (phacoemulsification) in 15 cases and after par plana vitrectomy (PPV) in 3 cases. Delayed-onset postoperative endophthalmitis occurred after trabeculectomy (*n* = 2), cataract surgery (*n* = 1) or keratoplasty (*n* = 1). Post-intravitreal injection endophthalmitis occurred in 7 cases following anti-VEGF agent administration and in 2 cases after dexamethasone implant injection.

The mean delay to diagnosis was 7.2 ± 5.9 days for acute postoperative endophthalmitis, 9.2 ± 6.6 months for delayed-onset postoperative endophthalmitis, 4.4 ± 2.7 days for endophthalmitis following intravitreal injection and 7.5 ± 6.4 days for cases related to open-globe injuries.

### Therapeutic management

All eyes received a combination of intravitreal and systemic antibiotic therapy at diagnosis. On average, patients underwent 2.8 ± 0.6 intravitreal antibiotic injections. In 10 patients, intravitreal antibiotics were modified for subsequent injections based on microbiological findings, while the remaining 40 patients received three intravitreal injections of vancomycin and cefazolin. Intravitreal dexamethasone was administered in 21 cases. Pars plana vitrectomy (PPV) was performed in 21 patients within the first month following diagnosis, with a mean delay of 9.1 ± 8.2 days, (range1 -28 days). No eye required evisceration.

### Sample characteristics

Among the 50 patients included in the study, an anterior chamber paracentesis was performed in 41 cases (92%) and a vitreous tap in 30 cases (60%). Both an anterior chamber paracentesis and a vitreous tap were performed in 21 cases (42%).

### Diagnostic yield of PCR versus culture

Among the 41 aqueous humor samples, conventional culture identified a pathogen in 9.8% of cases, compared to 26.8% for PCR (*p* = 0.016) and 26.8% for the combination of culture and PCR (*p* = 0.016).

Among the 30 vitreous samples, culture allowed bacterial identification in 36.7% of cases, compared to 56.7% for PCR (*p* = 0.109) and 63.3% for the combination of culture and PCR (*p* = 0.008).

Across all intraocular samples (*n* = 71), culture allowed bacterial identification in 21.1% of cases, compared to 39.4% for PCR (*p* = 0.002) and 42.3% for the combination of both (*p* < 0.001). PCR was positive in 26.8% of culture-negative cases (15/56) whereas culture was positive in 4.7% of PCR-negative cases (2/43).

Among the 21 patients who underwent both anterior chamber and vitreous sampling, when combining the results of the two samples, culture allowed bacterial identification in 33.3% of cases, compared to 66.6% for PCR (*p* = 0.039) and 71.4% for the combination of both (*p* = 0.008). In this subgroup, vitreous sampling identified a pathogen in 62.5% of cases when aqueous humor results were negative (10/16), whereas aqueous humor sampling led to identification in only 14.3% of cases with a negative vitreous result (1/7).

Among the 50 patients included in the study, when combining the results of the two samples for the 21 patients who underwent both anterior chamber and vitreous sampling, culture allowed bacterial identification in 28% of cases (14/50), compared to 48% for PCR (24/50, *p* = 0.021) and 54% for the combination of culture and PCR (27/50, *p* < 0.001) (Table 1, Additional file 4).

**Table 1:** Positivity of culture, PCR and the combination of both for aqueous humor and vitreous samples

	Aqueous humor (*n* = 41)	Vitreous (*n* = 30)	Aqueous humor and vitreous (*n* = 21)	Aqueous humor or vitreous (*n* = 71)
Culture +	4	11	7	15
PCR +	11	17	14	28
Culture or PCR +	11	19	15	30

PCR = polymerase chain reaction

### Bacterial ecology

A causative microorganism was identified in 35 (70%) cases (Table 2, Additional file 4). For 8 cases, both aqueous humor and vitreous were negative and identification was obtained either by blood culture (5 cases) or by corneal scraping (3 cases). When both culture and PCR were positive, the identified pathogens were fully concordant. Similarly, perfect agreement was observed between aqueous humor and vitreous samples when both yielded a result.

**Table 2:** Results of eubacterial PCR and/or cultures on vitreous and/or aqueous humor samples of the 50 cases of endophthalmitis

	16S rDNA PCR –	16S rDNA PCR +
Culture – **Total cases** Pathogen (number of cases)	**23** *S. aureus* (2)*; *S. infantarius* (1)*; *S. constellatus* (1)*; *H. influenzae* (1)*; *M. nonliquefaciens* (1)*; *E. coli* (1)*; *C. albicans* (1)* ; unknown (15)	**13** *S. epidermidis* (4); *S. saccharolyticus* (2); *S. aureus* (1); *S. pneumoniae* (1); *S. mitis* (1); *Clostridium* spp (1); *E. faecalis* (1); *E. coli* (1); *P. aeruginosa* (1)
Culture + **Total cases** Pathogen (number of cases)	**3** *C. albicans* (2); *E. faecalis* (1)	**11** *S. epidermidis* (5); *E. faecalis* (2); *S. pasteuri* (1); *S. oralis* (1); H. *influenzae* (1); *E. coli* (1)

PCR = polymerase chain reaction; spp = species

* Identification obtained through corneal scraping or blood cultures

Among the 35 identified cases, 16 different species were isolated: 71.4% (*n* = 25) were Gram-positive bacteria, 20% (*n* = 7) were Gram-negative, and 3 cases involved *Candida albicans*. The most frequently isolated species was *Staphylococcus epidermidis*, identified in 9 cases, followed by *Enterococcus faecalis* (*n* = 4), *Staphylococcus aureus* (*n* = 3), *Escherichia coli* (*n* = 3), *Candida albicans* (*n* = 3), *Staphylococcus saccharolyticus* (*n* = 2) and *Haemophilus influenzae* (*n* = 2). Nine other species were isolated in only one eye.

Antibiotic susceptibility testing (AST) was performed in 11 (22%) cases. Among the 14 culture-positive cases, AST was not available in three cases, including two isolates identified as *Candida albicans* and one isolate identified as *Staphylococcus epidermidis*. AST results were therefore available for 11 bacterial isolates, including nine Gram-positive and two Gram-negative bacteria. Vancomycin susceptibility was assessed only for Gram-positive isolates, all of which were susceptible. Two strains were resistant to fluoroquinolones (18.2%), and one showed intermediate susceptibility (9.1%). Susceptibility to cephalosporins and carbapenems was not assessed.

### Prognosis assessment

Residual BCVA after a minimum follow-up of 6 months was available for 26 patients, with a mean follow-up duration of 25.6 ± 18 months (range: 7–61 months). The overall mean BCVA was 1.3 ± 1.2 logMAR and 53.8% of patients had quantifiable visual acuity. Three eyes (11.5%) had BCVA limited to counting fingers, and 7 eyes (27%) had no perception of light.

BCVA was quantifiable in 62.5% of cases without pathogen identification, compared to 50% when a microorganism was identified (*p* = 0.424). Similarly, BCVA was quantifiable in 61.1% of culture-negative cases compared to 50% of culture-positive cases (*p* = 0.057), and in 56.3% of PCR-positive cases compared to 50% of PCR-negative cases (*p* = 0.774).

## Discussion

This study assessed the diagnostic performance of broad-range 16S rRNA gene PCR for the identification of pathogens in patients with presumed infectious endophthalmitis, in comparison and in combination with conventional microbiological culture. Our results demonstrate a clear improvement in microbiological yield when PCR is incorporated into the diagnostic workflow, particularly for vitreous samples and in culture-negative cases. These findings reinforce the complementary role of molecular techniques in enhancing diagnostic sensitivity in the context of endophthalmitis.

The overall identification rate in our series was 28% with culture alone, increasing to 48% with PCR and reaching 54% when both methods were combined. Although these identification rates were slightly lower than those reported in previous studies (ranging from 27% to 48% for culture and 42% to 82% for PCR), there was a clear improvement in diagnostic yield when PCR was added to culture [4, 5, 14, 15]. Notably, PCR enabled identification in 26.8% of culture-negative samples, which lies within the range described in the literature (18% to 52%) [4, 5, 15]. These results emphasize the added value of molecular techniques in overcoming the limitations of conventional microbiology, especially in cases where bacterial viability is compromised by prior antibiotic therapy or sample storage conditions.

A key finding of our study was the marked disparity in diagnostic yield between aqueous humor and vitreous samples. The identification rate from vitreous specimens was nearly twice that of aqueous humor, both for culture (36.7% vs. 9.8%) and PCR (56.7% vs. 26.8%). The superiority of vitreous samples has already been established in the literature [5, 14]. It is likely multifactorial and can be attributed to the biological characteristics of the sampled fluids. First, the vitreous body offers a larger sampling volume and provides a more permissive environment for bacterial proliferation. Second, the posterior segment is more directly affected in most cases of endophthalmitis, particularly in acute presentations. Conversely, aqueous humor is a relatively acellular and protein-poor medium with limited bacterial burden, and its sampling is often constrained by volume and safety considerations. These results support the preferential use of vitreous sampling when feasible. However, in our experience, obtaining undiluted vitreous specimens using a 25G or 23G needle can be challenging, due to the viscous consistency of the vitreous, which may clog the sampling needle. Moreover, the risk of iatrogenic complications, especially in phakic eyes, or limited operator experience may discourage its use, particularly outside of retinal surgical settings. In such cases, anterior chamber paracentesis remains a practical alternative, albeit with lower sensitivity.

The added diagnostic value of combining PCR and culture was particularly evident in the subset of patients who underwent both anterior chamber and vitreous sampling. In this group, culture alone identified pathogens in 33.3% of cases, while PCR identified them in 66.6%. The combination of both techniques increased the identification rate to 71.4%, which is consistent with prior findings in the literature [4, 5, 15]. Notably, the vitreous sample identified a pathogen in 62.5% of cases where the aqueous sample was negative, whereas the contrary was only true in 14.3% of cases. These findings confirm the higher diagnostic utility of vitreous samples and underscore the benefit of pairing molecular and conventional diagnostics in the same specimen.

The microbial spectrum observed in our study was consistent with previous literature [12, 14, 18]. Gram-positive organisms accounted for over two-thirds of identified cases, with *Staphylococcus epidermidis* being the most frequently isolated species. Gram-negative bacteria represented 20% of isolates and *Candida albicans* was identified in three cases of endogenous endophthalmitis. Full concordance was observed between culture and PCR results when both were positive, which confirms the reliability of molecular techniques for species-level identification. These results validate PCR as a credible diagnostic tool in this clinical setting.

One potential limitation of 16 S rRNA PCR is its inability to provide antibiotic susceptibility data, which remains essential for tailored antimicrobial therapy. However, in our series, resistance to commonly used intravitreal antibiotics was infrequent [19]. No resistance was observed to vancomycin, and fluoroquinolone resistance was limited to a small number of cases (2 out of 11 tested isolates). In such settings, pathogen identification alone can guide a “semi-adapted” empirical treatment, considering intrinsic resistance profiles of the species involved. This approach could be particularly relevant in ophthalmology, where available antibiotic options are limited and resistance remains uncommon.

We also investigated whether the identification of a pathogen had an impact on visual outcomes. Contrary to expectations, no significant association was found between bacterial identification and improved best-corrected visual acuity (BCVA). In fact, a trend toward worse visual outcomes in culture-positive cases was observed. This paradox has been previously described and may be explained by the “inoculum effect”, whereby positive culture reflects a higher bacterial load and thus a more aggressive infection [14, 20]. It is also plausible that clinical management remains unchanged despite microbiological findings, given the empirical nature of standard treatment protocols. Indeed, antibiotic therapy is often initiated empirically at the time of diagnosis and is rarely modified even when microbiological results become available. Moreover, intraocular inflammation, which can progress independently of bacterial viability, remains a key determinant of visual prognosis [20]. These findings highlight the complexity of endophthalmitis management, where both infectious and inflammatory mechanisms contribute to visual deterioration. While rapid and accurate pathogen identification is valuable, it may not directly translate into improved outcomes unless it is paired with timely adjustments in therapy and optimized anti-inflammatory strategies. In addition, anti-inflammatory management strategies may differ between centers and could influence outcomes. While intravitreal or subconjunctival corticosteroids are commonly used in France and recommended by French guidelines [19], high-dose systemic corticosteroid therapy has been proposed as an alternative approach. Although it may provide a more global modulation of inflammation and avoid additional intraocular injections, it results in lower intravitreal concentrations compared with intravitreal or periocular injections [21] and is not without risks, particularly in fragile or elderly patients.

This study has nevertheless some limits. The retrospective design of our study introduces inherent limitations, including potential selection bias and incomplete follow-up data. Furthermore, the relatively small sample size and the monocentric design of this study limits the statistical power of subgroup analyses, particularly regarding visual prognosis. Nevertheless, our study has important strengths. The 7-year inclusion period aligns with the consistent use of PCR in our institution, ensuring homogeneity in diagnostic methodology and minimizing selection bias related to evolving microbiological practices. Additionally, the inclusion of both exogenous and endogenous cases provides a comprehensive view of microbial profiles across clinical scenarios.

## Conclusion

This study confirms the significant added value of broad-range 16S rRNA gene PCR in the microbiological diagnosis of infectious endophthalmitis. When combined with conventional culture, PCR substantially increases the overall identification rate, particularly for vitreous samples and in culture-negative cases. Although it does not provide antimicrobial susceptibility data, PCR remains a reliable and sensitive diagnostic tool. Its rapid and accurate identification capacity supports more rational antimicrobial use and reduces diagnostic uncertainty, particularly in complex or partially treated infections. Integrating molecular techniques alongside conventional methods yields optimal diagnostic performance and may enhance clinical decision-making.

Future prospective studies should assess the impact of PCR-guided therapy on visual outcomes and evaluate the cost-effectiveness of systematic molecular testing to support its broader implementation in the management of ophthalmic infectious diseases.

## Supplementary Information

Below is the link to the electronic supplementary material.

Supplementary Material 1

## Data Availability

All data generated and analyzed during this study are included in this article and its supplementary information files.
