# Supplementary material for: Contribution of 16S rRNA gene PCR to microbiological diagnosis of endophthalmitis: a retrospective clinical cohort study of 71 vitreous and aqueous humor samples
Source: J Ophthalmic Inflamm Infect. 2026 Apr 25;16:24. doi: 10.1186/s12348-026-00581-2 (PMC13247020; doi:10.1186/s12348-026-00581-2)
Supplement: Supplementary file 1 — Supplementary Material 1 [file 12348_2026_581_MOESM1_ESM.docx]

# Additional files

Additional file 1. Conversion of visual acuity between the LogMAR scale and the Monoyer scale.

| LogMAR scale | Monoyer scale |
| --- | --- |
| 1,3 | 1/20 |
| 1 | 1/10 |
| 0,8 | 1,6/10 |
| 0,7 | 2/10 |
| 0,6 | 2,5/10 |
| 0,5 | 3/10 |
| 0,4 | 4/10 |
| 0,3 | 5/10 |
| 0,2 | 6/10 |
| 0,2 | 7/10 |
| 0,1 | 8/10 |
| 0,1 | 9/10 |
| 0 | 10/10 |

Additional file 2. Estimated LogMAR values for non-quantifiable visual acuities.

| LogMAR scale | Non-quantifiable visual acuities |
| --- | --- |
| 3 | No light perception |
| 2,6 | Light perception |
| 2,3 | Hand motion |
| 2 | Couting fingers at 30cm |
| 1,7 | Couting fingers at 1m |

Additional file 3. Clinical characteristics and treatment details of the study cohort.

| Patient ID | Sex | Age at diagnosis | Etiology | Side | Diabetes mellitus | Delay of diagnosis (days) | BCVA at diagnosis (LogMAR) | Residual BCVA (LogMAR) | Delay of residual BCVA (days) | PPV | Delay of PPV (days) | Intravitreal antibiotics | Number of intravitreal antibiotic injections | Number of intravitreal dexamethasone injections |
| --- | --- | --- | --- | --- | --- | --- | --- | --- | --- | --- | --- | --- | --- | --- |
| 44 | F | 61 | Endogenous endophthalmitis | OD | Yes |  | 1,3 |  |  | No |  | Other | 3 |  |
| 3 | M | 82 | Delayed-onset postoperative endophthalmitis (keratoplasty) | OD | No | 169 | 2,3 | 0,4 | 678 | No |  | VAN-CFZ | 3 |  |
| 18 | F | 87 | Infectious keratitis | OS | No |  | 2,3 | 3 |  | No |  | VAN-CFZ | 2 |  |
| 27 | F | 93 | Unknown | OS | No |  | 2,3 |  |  | No |  | VAN-CFZ | 3 |  |
| 16 | F | 78 | Acute postoperative endophthalmitis (cataract surgery) | OS | No | 5 | 0,5 |  |  | No |  | VAN-CFZ | 3 | 1 |
| 2 | F | 78 | Acute postoperative endophthalmitis (cataract surgery) | OD | No | 2 | 2,3 | 0,1 | 1798 | Yes | 9 | VAN-CFZ | 3 |  |
| 19 | M | 74 | Infectious keratitis | OS | No |  | 2,3 |  |  | No |  | VAN-CFZ | 2 |  |
| 26 | F | 87 | Infectious keratitis | OS | Yes |  | 2,6 | 3 |  | No |  | VAN-CFZ | 2 |  |
| 28 | M | 82 | Endogenous endophthalmitis | OD | Yes |  | 0,5 |  |  | No |  | Other | 1 |  |
| 29 | M | 60 | Endogenous endophthalmitis | OS | Yes |  | 2 |  |  | No |  | No |  |  |
| 30 | F | 69 | Acute postoperative endophthalmitis (cataract surgery) | OS | No | 4 | 2,3 | 0,1 | 645 | Yes | 1 | VAN-CFZ | 3 | 1 |
| 11 | F | 79 | Intravitreal injection (anti-VEGF) | OS | No | 1 | 2,3 |  |  | Yes | 10 | Other | 3 | 2 |
| 4 | M | 48 | Endogenous endophthalmitis | OS | No |  | 2 |  |  | Yes | 23 | VAN-CFZ | 2 |  |
| 45 | M | 82 | Endogenous endophthalmitis | OS | No |  | 2,3 |  |  | No |  | VAN-CFZ | 3 |  |
| 10 | M | 59 | Unknown | OS | No |  | 2,3 | 3 | 1651 | No |  | VAN-CFZ | 3 |  |
| 50 | F | 74 | Endogenous endophthalmitis | OS | Yes |  | 2,3 |  |  | No |  | Other | 2 |  |
| 33 | M | 78 | Endogenous endophthalmitis | OD | No |  | 2 |  |  | No |  | Other | 3 |  |
| 23 | M | 75 | Endogenous endophthalmitis | OD | No |  | 2,3 |  |  | No |  | VAN-CFZ | 3 |  |
| 48 | M | 46 | Open-globe injury | OS | No |  | 2,6 |  |  | Yes | 4 | Other | 2 |  |
| 20 | M | 58 | Open-globe injury | OD | No | 12 | 2 | 2 | 703 | No |  | VAN-CFZ | 3 | 1 |
| 36 | M | 29 | Open-globe injury | OS | No | 3 | 0,8 | 0 | 276 | No |  | VAN-CFZ | 3 |  |
| 42 | F | 77 | Intravitreal injection (anti-VEGF) | OD | No | 3 | 2 |  |  | No |  | VAN-CFZ | 3 | 2 |
| 1 | F | 79 | Delayed-onset postoperative endophthalmitis (blebitis) | OD | No | 511 | 0,5 | 0 | 517 | No |  | VAN-CFZ | 3 |  |
| 39 | F | 91 | Intravitreal injection (dexamethasone implant) | OS | No | 3 | 2,3 |  |  | No |  | VAN-CFZ | 3 | 2 |
| 40 | M | 73 | Acute postoperative endophthalmitis (cataract surgery) | OS | Yes | 11 | 2,3 |  |  | Yes | 17 | VAN-CFZ | 3 | 2 |
| 41 | F | 78 | Intravitreal injection (anti-VEGF) | OS | No | 4 | 2,3 | 1,3 | 391 | Yes | 28 | VAN-CFZ | 3 | 1 |
| 37 | F | 49 | Intravitreal injection (anti-VEGF) | OD | Yes | 4 | 2,3 | 0,4 | 440 | Yes | 24 | VAN-CFZ | 3 | 2 |
| 32 | M | 73 | Acute postoperative endophthalmitis (cataract surgery) | OD | No | 3 | 2,3 | 0,4 | 503 | No |  | VAN-CFZ | 3 |  |
| 21 | M | 68 | Delayed-onset postoperative endophthalmitis (cataract surgery) | OS | Yes | 160 | 0,3 | 0 | 1210 | No |  | VAN-CFZ | 3 |  |
| 49 | M | 58 | Acute postoperative endophthalmitis (cataract surgery) | OD | No | 7 | 2,3 |  |  | No |  | VAN-CFZ | 3 | 1 |
| 24 | F | 85 | Acute postoperative endophthalmitis (PPV) | OS | No | 6 | 2,3 | 3 | 658 | No |  | VAN-CFZ | 3 | 1 |
| 43 | F | 95 | Acute postoperative endophthalmitis (cataract surgery) | OD | No | 5 | 2,3 |  |  | Yes | 6 | VAN-CFZ | 3 |  |
| 14 | F | 80 | Acute postoperative endophthalmitis (PPV) | OS | No | 7 | 2 | 0,7 | 1640 | No |  | VAN-CFZ | 3 | 1 |
| 31 | F | 77 | Intravitreal injection (anti-VEGF) | OS | No |  | 2,3 | 0,2 | 260 | Yes | 2 | VAN-CFZ | 2 | 2 |
| 25 | F | 75 | Acute postoperative endophthalmitis (cataract surgery) | OD | No | 6 | 2,3 | 0,3 | 1260 | Yes | 7 | VAN-CFZ | 3 | 1 |
| 13 | F | 69 | Acute postoperative endophthalmitis (cataract surgery) | OS | Yes | 5 | 2,6 |  |  | Yes | 2 | VAN-CFZ | 3 |  |
| 9 | M | 26 | Open-globe injury | OD | No |  | 0,2 | 0,3 | 201 | Yes | 11 | VAN-CFZ | 3 |  |
| 8 | F | 84 | Intravitreal injection (anti-VEGF) | OD | No | 6 | 2 |  |  | Yes | 9 | VAN-CFZ | 3 |  |
| 34 | M | 79 | Endogenous endophthalmitis | OS | Yes |  | 2 | 1,7 | 496 | Yes | 17 | Other | 3 |  |
| 46 | M | 66 | Acute postoperative endophthalmitis (cataract surgery) | OS | No | 4 | 2,6 | 3 |  | Yes | 1 | VAN-CFZ | 4 |  |
| 35 | F | 60 | Acute postoperative endophthalmitis (cataract surgery) | OD | No | 10 | 2,3 | 2 | 384 | Yes | 1 | VAN-CFZ | 3 | 2 |
| 12 | M | 78 | Intravitreal injection (dexamethasone implant) | OS | No | 10 | 2 |  |  | Yes | 7 | Other | 3 | 2 |
| 7 | M | 50 | Acute postoperative endophthalmitis (PPV) | OD | No | 3 | 2,6 |  |  | No |  | VAN-CFZ | 2 |  |
| 17 | M | 90 | Acute postoperative endophthalmitis (cataract surgery) | OS | No | 7 | 2,3 |  |  | No |  | VAN-CFZ | 3 | 1 |
| 47 | M | 60 | Endogenous endophthalmitis | OS | No |  | 2,6 | 3 |  | No |  | Other | 1 |  |
| 5 | M | 71 | Acute postoperative endophthalmitis (cataract surgery) | OD | No | 12 | 2,6 | 0,4 | 1867 | Yes | 8 | VAN-CFZ | 3 | 1 |
| 38 | F | 88 | Acute postoperative endophthalmitis (cataract surgery) | OS | No | 28 | 2,3 |  |  | No |  | VAN-CFZ | 3 | 2 |
| 6 | M | 69 | Delayed-onset postoperative endophthalmitis (blebitis) | OS | No |  | 2,6 | 2 | 290 | Yes | 4 | VAN-CFZ | 3 | 1 |
| 15 | F | 86 | Intravitreal injection (anti-VEGF) | OD | No | 4 | 3 | 3 |  | No |  | VAN-CFZ | 3 |  |
| 22 | M | 42 | Acute postoperative endophthalmitis (cataract surgery) | OD | No | 4 | 2,3 | 1 | 472 | Yes | 2 | VAN-CFZ | 3 | 1 |
| F = female ; M = male ; VEGF = vascular endothelial growth factor ; OD = oculus dexter ; OS = oculus sinister ; BCVA = best corrected visual acuity ; PPV = pars plana vitrectomy ; VAN = vancomycin ; CFZ = cefazolin. | | | | | | | | | | | | | | |

Additional file 4. Microbiological findings of the study cohort.

| Patient ID | Sex | Age at diagnosis | Identification | Gram | Species | Aqueous humor samples (culture and PCR combined) | Vitreous samples (culture and PCR combined) | Culture results (aqueous humor and vitreous samples) | Culture results (aqueous humor samples only) | Culture results (vitreous samples only) | PCR results (aqueous humor and vitreous samples) | PCR results (aqueous humor samples only) | PCR results (vitreous samples only) | Antibiotic Susceptibility Testing |
| --- | --- | --- | --- | --- | --- | --- | --- | --- | --- | --- | --- | --- | --- | --- |
| 44 | F | 61 | Yes | Yeast | *Candida albicans* | Negative | Negative | Negative | Negative | Negative | Negative | Negative | Negative | No |
| 3 | M | 82 | No | Unknown | Unknown | Negative | Negative | Negative | Negative | Negative | Negative | Negative | Negative | No |
| 18 | F | 87 | No | Unknown | Unknown | Negative | Negative | Negative | Negative | Negative | Negative | Negative | Negative | No |
| 27 | F | 93 | No | Unknown | Unknown | Negative | Negative | Negative | Negative | Negative | Negative | Negative | Negative | No |
| 16 | F | 78 | No | Unknown | Unknown | Negative | Negative | Negative | Negative | Negative | Negative | Negative | Negative | No |
| 2 | F | 78 | No | Unknown | Unknown | Negative | Negative | Negative | Negative | Negative | Negative | Negative | Negative | No |
| 19 | M | 74 | Yes | Negative | *Moraxella nonliquefaciens* | NP | Negative | Negative | NP | Negative | Negative | NP | Negative | Yes |
| 26 | F | 87 | Yes | Positive | *Staphylococcus aureus* | NP | Negative | Negative | NP | Negative | Negative | NP | Negative | Yes |
| 28 | M | 82 | No | Unknown | Unknown | NP | Negative | Negative | NP | Negative | Negative | NP | Negative | No |
| 29 | M | 60 | Yes | Yeast | *Candida albicans* | Positive | Negative | Positive | Positive | Negative | Negative | NP | Negative | No |
| 30 | F | 69 | Yes | Positive | *Enterococcus faecalis* | Positive | Negative | Negative | Negative | Negative | Positive | Positive | Negative | No |
| 11 | F | 79 | Yes | Positive | *Enterococcus faecalis* | Negative | Positive | Positive | Negative | Positive | Negative | Negative | Negative | Yes |
| 4 | M | 48 | Yes | Yeast | *Candida albicans* | Negative | Positive | Positive | Negative | Positive | Negative | NP | Negative | No |
| 45 | M | 82 | Yes | Negative | *Escherichia coli* | Negative | NP | Negative | Negative | NP | Negative | Negative | NP | Yes |
| 10 | M | 59 | Yes | Negative | *Haemophilus influenzae* | Negative | NP | Negative | Negative | NP | Negative | Negative | NP | No |
| 50 | F | 74 | Yes | Positive | *Staphylococcus aureus* | Negative | NP | Negative | Negative | NP | Negative | Negative | NP | Yes |
| 33 | M | 78 | Yes | Positive | *Streptococcus constellatus* | Negative | NP | Negative | Negative | NP | Negative | Negative | NP | Yes |
| 23 | M | 75 | Yes | Positive | *Streptococcus infantarius* | Negative | NP | Negative | Negative | NP | Negative | Negative | NP | Yes |
| 48 | M | 46 | No | Unknown | Unknown | Negative | NP | Negative | Negative | NP | Negative | Negative | NP | No |
| 20 | M | 58 | No | Unknown | Unknown | Negative | NP | Negative | Negative | NP | Negative | Negative | NP | No |
| 36 | M | 29 | No | Unknown | Unknown | Negative | NP | Negative | Negative | NP | Negative | Negative | NP | No |
| 42 | F | 77 | No | Unknown | Unknown | Negative | NP | Negative | Negative | NP | Negative | Negative | NP | No |
| 1 | F | 79 | No | Unknown | Unknown | Negative | NP | Negative | Negative | NP | Negative | Negative | NP | No |
| 39 | F | 91 | No | Unknown | Unknown | Negative | NP | Negative | Negative | NP | Negative | Negative | NP | No |
| 40 | M | 73 | No | Unknown | Unknown | Negative | NP | Negative | Negative | NP | Negative | Negative | NP | No |
| 41 | F | 78 | No | Unknown | Unknown | Negative | NP | Negative | Negative | NP | Negative | Negative | NP | No |
| 37 | F | 49 | No | Unknown | Unknown | Negative | NP | Negative | Negative | NP | Negative | Negative | NP | No |
| 32 | M | 73 | Yes | Negative | *Escherichia coli* | Positive | NP | Negative | Negative | NP | Positive | Positive | NP | No |
| 21 | M | 68 | Yes | Positive | *Clostridium sp* | Positive | NP | Negative | Negative | NP | Positive | Positive | NP | No |
| 49 | M | 58 | Yes | Positive | *Staphylococcus epidermidis* | Positive | NP | Negative | Negative | NP | Positive | Positive | NP | No |
| 24 | F | 85 | Yes | Positive | *Staphylococcus epidermidis* | Positive | NP | Negative | Negative | NP | Positive | Positive | NP | No |
| 43 | F | 95 | Yes | Positive | *Enterococcus faecalis* | Positive | NP | Positive | Positive | NP | Positive | Positive | NP | Yes |
| 14 | F | 80 | Yes | Positive | *Staphylococcus epidermidis* | Positive | NP | Positive | Positive | NP | Positive | Positive | NP | No |
| 31 | F | 77 | Yes | Positive | *Staphylococcus aureus* | Negative | Positive | Negative | Negative | Negative | Positive | Negative | Positive | No |
| 25 | F | 75 | Yes | Positive | *Staphylococcus epidermidis* | Negative | Positive | Negative | Negative | Negative | Positive | Negative | Positive | No |
| 13 | F | 69 | Yes | Positive | *Staphylococcus epidermidis* | Negative | Positive | Negative | Negative | Negative | Positive | Negative | Positive | No |
| 9 | M | 26 | Yes | Positive | *Staphylococcus saccharolyticus* | Negative | Positive | Negative | Negative | Negative | Positive | Negative | Positive | No |
| 8 | F | 84 | Yes | Positive | *Staphylococcus saccharolyticus* | Negative | Positive | Negative | Negative | Negative | Positive | Negative | Positive | No |
| 34 | M | 79 | Yes | Positive | *Streptococcus pneumoniae* | Negative | Positive | Negative | Negative | Negative | Positive | Negative | Positive | Yes |
| 46 | M | 66 | Yes | Negative | *Pseudomonas aeruginosa* | NP | Positive | Negative | NP | Negative | Positive | NP | Positive | No |
| 35 | F | 60 | Yes | Positive | *Streptococcus mitis* | Positive | Positive | Negative | Negative | Negative | Positive | Positive | Positive | No |
| 12 | M | 78 | Yes | Positive | *Staphylococcus epidermidis* | Negative | Positive | Positive | Negative | Positive | Positive | Negative | Positive | Yes |
| 7 | M | 50 | Yes | Positive | *Staphylococcus epidermidis* | Negative | Positive | Positive | Negative | Positive | Positive | Negative | Positive | Yes |
| 17 | M | 90 | Yes | Positive | *Staphylococcus epidermidis* | Negative | Positive | Positive | Negative | Positive | Positive | Negative | Positive | Yes |
| 47 | M | 60 | Yes | Negative | *Escherichia coli* | Negative | Positive | Positive | Negative | Positive | Positive | NP | Positive | Yes |
| 5 | M | 71 | Yes | Positive | *Staphylococcus epidermidis* | Negative | Positive | Positive | Negative | Positive | Positive | NP | Positive | Yes |
| 38 | F | 88 | Yes | Positive | *Staphylococcus pasteuri* | NP | Positive | Positive | NP | Positive | Positive | NP | Positive | Yes |
| 6 | M | 69 | Yes | Negative | *Haemophilus influenzae* | Positive | Positive | Positive | Negative | Positive | Positive | Positive | Positive | Yes |
| 15 | F | 86 | Yes | Positive | *Enterococcus faecalis* | Positive | Positive | Positive | Positive | Positive | Positive | Positive | Positive | Yes |
| 22 | M | 42 | Yes | Positive | *Streptococcus oralis* | Positive | Positive | Positive | Positive | Positive | Positive | Positive | Positive | Yes |
| PCR = polymerase chain reaction ; NP = not performed. | | | | | | | | | | | | | | |
